# Newly developed plastic stent delivery system for endoscopic ultrasonography-guided gallbladder drainage: Experiments on gallbladder models

**DOI:** 10.1055/a-2734-0383

**Published:** 2025-11-11

**Authors:** Tesshin Ban, Yoshimasa Kubota, Takashi Joh

**Affiliations:** 1 36884 Department of Gastroenterology and Hepatology, Gamagori City Hospital, Gamagori, Japan

## Abstract

Endoscopic ultrasonography-guided gallbladder drainage (EUS-GBD) using the current plastic stent (PS) system requires multiple steps for stent deployment and carries the risk of bile leakage from the puncture site. In this study, we developed a single-step PS system that includes a 3F electrocautery metal tip on the inner sheath mounted with a 7F/4-cm double-pigtail PS and experimentally evaluated it on a gallbladder model mimicking EUS-GBD. We assessed procedure time, intra-gallbladder pressure-drop ratios, and bile leakage using a newly developed single-step PS system, comparing it with the current PS system during drainage attempts on a gallbladder model. Mean duration times for the three attempts were 2 minutes 59 seconds and 27 seconds for the current PS system and the newly developed PS system groups, respectively ( *P* < 0.001). Mean intra-gallbladder pressure-drop ratios were 86.7% in the current PS system group and 7.6% in the newly developed PS system group ( *P* < 0.001). The newly developed PS system prevented bile leakage from the puncture site. The newly developed PS system saved significant time, maintained intra-gallbladder pressure, and prevented bile leakage during the procedure when compared with the current PS system.

## Introduction

Endoscopic ultrasonography-guided gallbladder drainage (EUS-GBD) is recommended over standard percutaneous or transpapillary approaches in fragile patients with acute cholecystitis 1 2 . In this clinical scenario, EUS-GBD using an electrocautery-enhanced lumen-apposing metallic stent (EC-LAMS) is preferred over plastic stents (PS) because of its simpler procedural steps and fewer adverse events (AEs) 1 2 .

We hypothesized that a single-step PS delivery system for EUS-GBD would reduce incidence of AEs, particularly bile leakage 3 4 5 . To test this hypothesis, we conducted a preliminary experimental study simulating EUS-GBD using a gallbladder model and a remnant porcine gallbladder obtained from meat processing and compared its performance with that of a commercially available PS.

## Methods

### Aim and study design

This preliminary experimental study simulated EUS-GBD to compare a single-step PS delivery system with the current PS system using a gallbladder model and a porcine gallbladder.

### Prepared gallbladder and experimental system

EUS-GBD experiment using the current PS and a newly developed single-step PS system for artificial and porcine gallbladders.Video 1

**Fig. 1:**
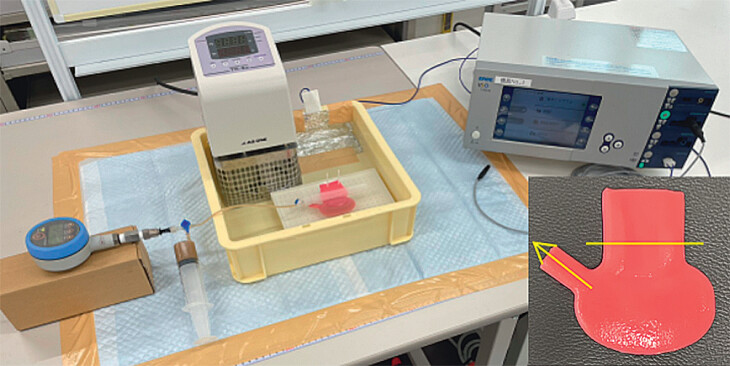
Experimental system. At the center, a gallbladder model is immersed in a 0.18 wt% saline solution maintained at 36.5°C by the incubator. The gallbladder model is sealed with a clip at the yellow line and connected to a manometer and an injection syringe (yellow arrow). On the left, the zero-calibrated gallbladder is connected to a manometer and a syringe filled with a brown-colored 2.0 wt%, 13–18 mPa∙ methylcellulose solution. On the right side, the incubator was connected to a neutral pad and interfaced with an electrosurgical generator.

The experimental setup is shown in ( Fig. 1 , Video 1 ). First, a spheroidal gallbladder model was constructed from 3 mm-thick polyvinyl alcohol artificial membrane with a major radius of 55 mm and a minor radius of 40 mm. The model was immersed in a 0.18 wt% saline solution maintained at 36.5°C using an incubator. Subsequently, the gallbladder was connected to a manometer to establish zero-calibration of intra-gallbladder pressure. Then, 50 mL of brown-colored 2.0 wt%, 13–18 mPa∙s methylcellulose solution was injected into the gallbladder. Intra-gallbladder pressure was continuously monitored from the point of puncture until stent deployment. The incubator, attached to a neutral pad, was connected to an electrosurgical generator (VIO300D, ERBE, Tübingen, Germany) set to the monopolar mode with Endo Cut 1 and Effect 2.

In addition, a residual porcine gallbladder obtained from meat processing was used to examine bile leakage following drainage using a single-step PS delivery system.

### Gallbladder drainage

**Fig. 2:**
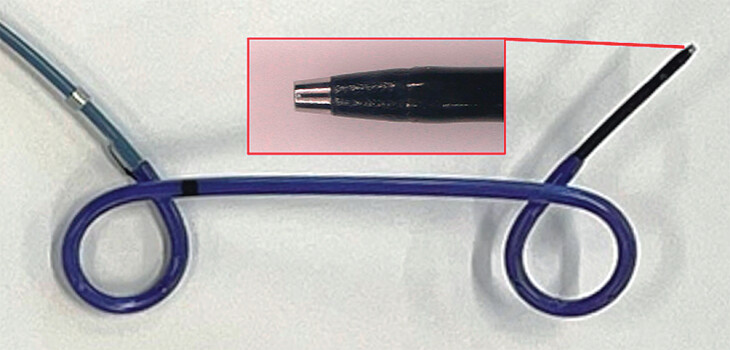
Newly developed PS system. The newly developed PS system includes a tapered inner sheath with a 3F metal tip for electrocautery dilatation. The guidewire was passed through the inner sheath. A double-pigtail PS (7F/4 cm) connected by a thread to the pusher sheath was mounted on the inner sheath. The inner sheath disconnects the PS from the pusher sheath. PS, plastic stent.

Simulating EUS-GBD with the current PS system (Advanix J; Boston Scientific, Marlborough, Massachusetts, United States) required multiple steps: puncture using a 19G lancet needle, coiling with a 0.025-inch hydrophilic guidewire, removal of the needle, electrocautery dilation (6F Cysto-Gastro-Sets; Endoflex GmbH, Voerde, Germany), delivery of a 7F/4-cm double-pigtailed PS, and removal of the inner sheath 4 5 . The newly developed PS system (Japan Lifeline, Tokyo, Japan) was composed of a tapered inner sheath with a 3F metal tip, which was equipped with electrocautery dilation. The guidewire was passed through the inner sheath. A double-pigtail PS (7F/4 cm) connected by a thread to the pusher sheath was mounted on the inner sheath. Withdrawal of the inner sheath released the PS by detaching the thread from it. This system enabled a single-step PS delivery without device exchange over the guidewire, and drainage was performed in the following sequence: electrocautery puncture, guidewire advancement, and PS deployment ( Fig. 2 , Video 1 ). In both experiments, manual puncture-to-stent deployment was performed with as vertical an orientation as possible against the walls.

### Outcome measures

Outcome measures included procedure time from puncture to PS deployment during gallbladder drainage in the artificial gallbladder model and the ratio of the intra-gallbladder pressure drop. The ratio of intra-gallbladder pressure drop was calculated as (ΔP/ pre-puncture pressure) × 100. ΔP was defined as the difference between the intra-gallbladder pressure at the time of puncture and the pressure at stent deployment. This procedure was repeated three times for both current and single-step PS systems. All procedures were recorded and analyzed using video footage. Bile leakage was observed at the puncture site. In addition, a video of bile leakage from the porcine gallbladder following gallbladder drainage using the single-step PS delivery system was recorded.

### Statistical analysis

Procedure time and ratio of intra-gallbladder pressure drop were expressed as means with standard deviations (SDs). Statistical analyses were performed using Student’s *t* -test with the IBM statistical package for social sciences statistics 28 (IBM Japan, Ltd., Tokyo, Japan), and statistical significance was set at *P* < 0.05. Finally, a post-hoc power analysis was performed using G*Power software ( https://www.psychologie.hhu.de/arbeitsgruppen/allgemeine-psychologie-und-arbeitspsychologie/gpower ). We entered each mean and SD to calculate the effect size (Cohen's d). After that, the statistical power (1–β error) was calculated using an α error of 0.05, a sample size of three, and the obtained effect size.

## Results

### Time from puncture to PS deployment during gallbladder drainage

**Fig. 3:**
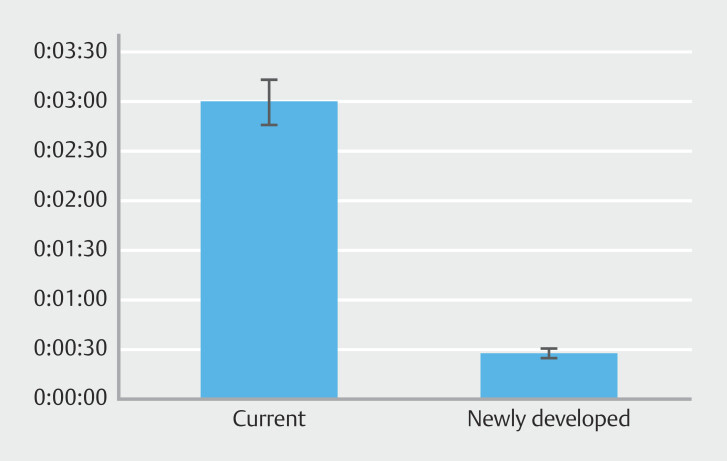
Time from puncture to PS deployment during gallbladder drainage. The newly developed PS system saved significantly more time (mean, 27 s; SD, 3 s) than the current PS system (mean, 2 min, 59 s; SD, 14 s). PS, plastic stent; SD, standard deviation.

**Fig. 4:**
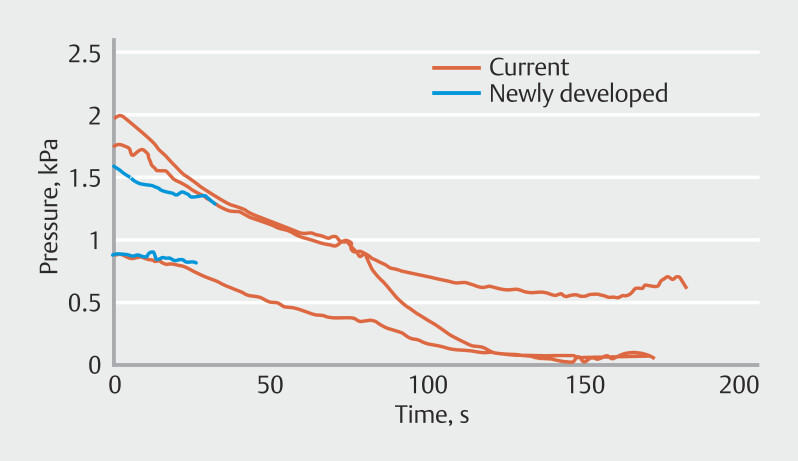
Intra-gallbladder pressure during gallbladder drainage. The newly developed PS system quickly completes the drainage process, causing a slight decrease in the intra-gallbladder pressure. In contrast, the current PS system takes longer, resulting in a sharp decrease in pressure. Notably, the pressure drop accelerated further after dilation (around the midpoint of the orange lines).

Table 1 summarizes procedure time from puncture to PS deployment during gallbladder drainage using an artificial gallbladder model. Mean duration times for the three attempts were 2 min 59 s and 27 s for the current PS system and the newly developed PS system groups, respectively ( *P* < 0.001). The newly developed PS system was significantly more time efficient ( Fig. 3 , Fig. 4 ) with a statistical power of 1.0.

**Table:** **Table 1** Time from gallbladder puncture to PS deployment during gallbladder drainage.

**PS system**	**Attempt**	**Procedure time**	**Mean (SD)**	***P* value **
Current	1	3 min 16 s	2 min 59 s (14 s)	< 0.001
	2	2 min 55 s		
	3	2 min 47 s		
Newly developed	1	31 s	27 s (3 s)	
	2	25 s		
	3	24 s		
PS, plastic stent; SD, standard deviation.				

### Intra-gallbladder pressures during gallbladder drainage

**Fig. 5:**
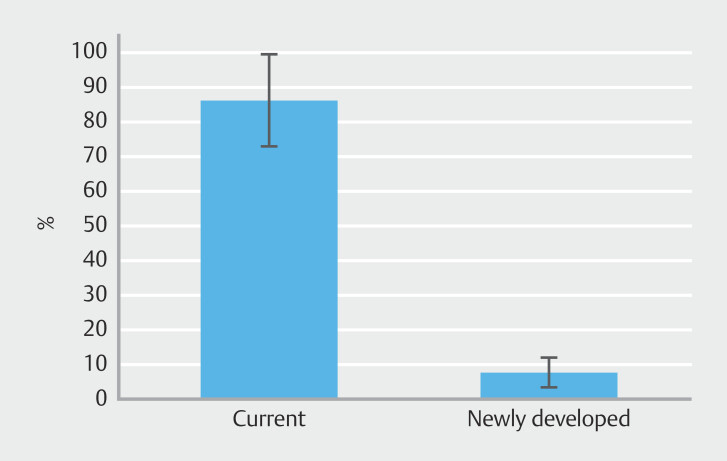
Intra-gallbladder pressure drop ratio during gallbladder drainage. The newly developed PS system (mean, 7.6%; SD, 4.4%) demonstrated a significantly lower intra-gallbladder pressure drop ratio than the current PS system (mean, 86.7%; SD, 13.6%).

Table 2 summarizes intra-gallbladder pressures, including pre-puncture, pre-stent deployment, ΔP, and pressure drop ratio during gallbladder drainage for the artificial gallbladder model across three attempts. Mean pressure drop ratios were 86.7% and 7.6% for the current PS system and newly developed PS system groups, respectively ( *P* < 0.001). The newly developed PS system was significantly effective in maintaining intra-gallbladder pressure ( Fig. 4 , Fig. 5 ) with a statistical power of 0.99.

**Table:** **Table 2** Intra-gallbladder pressures during gallbladder drainage.

**PS system**	**Attempt**	**Pre-puncture, kPa**	**Pre-deployment, kPa**	**ΔP, kPa**	**Pressure drop ratio, %**	**Mean, % (SD)**	***P* value **
Current	1	1.97	0.56	1.41	71.6	86.7 (13.6)	< 0.001
	2	1.76	0.04	1.72	97.7		
	3	0.88	0.08	0.80	90.9		
Newly developed	1	1.58	1.38	0.20	12.7	7.6 (4.4)	
	2	0.87	0.83	0.04	4.6		
	3	0.89	0.84	0.05	5.6		
PS, plastic stent; SD, standard deviation.							

### Bile leakage from the puncture site on the gallbladder

During gallbladder drainage using the current PS system, the artificial gallbladder shrinks due to continuous bile leakage from the puncture site because a multistep procedure is required for stent deployment. As a result, the PS loses its drainage effect ( Video 1 ). In contrast, the artificial gallbladder retained its shape throughout gallbladder drainage with the newly developed PS system, and bile was efficiently drained only from the PS ( Video 1 ). Similarly, during drainage of the porcine gallbladder using the newly developed PS system, the gallbladder maintained its shape, and bile was gradually drained from the PS ( Video 1 ).

### Discussion

In this study, we compared a single-step PS delivery system with the current PS system in experimental EUS-GBD and observed the following outcomes. First, the single-step PS delivery system significantly reduced procedure time, and second, it could potentially minimize bile leakage during EUS-GBD. EUS-GBD with the current PS system requires multiple steps after needle puncture for PS deployment, including needle exchange with a dilator and exchange of the dilator with the PS. Therefore, as expected, the single-step PS system saves time. As shown in Video 1, bile leakage occurred at the gallbladder puncture site during device exchange when the current PS system was used for drainage. In contrast, the single-step PS system prevented bile leakage from the puncture site and maintained intra-gallbladder pressure until stent deployment. This may prevent bile-induced peritonitis and ensure effective gallbladder drainage into the alimentary tract through a stent after EUS-GBD. Recently, we demonstrated that the inner sheath of a PS delivery system can aspirate and lavage infectious bile from the gallbladder 6 . Therefore, this single-step PS system with gallbladder lavage may provide technical and clinical success rates comparable to those of EUS-GBD with EC-LAMS for managing patients with acute cholecystitis. However, risk of bile leakage remains despite the potential of 7F PS to seal the puncture site following 3F electrocautery dilation. The risk arises because excessive cauterization may enlarge the puncture site beyond expectation. Therefore, electrocautery dilation should be completed as quickly as possible to minimize risk of excessive electrocauterization. Optimal electrocautery settings should be explored further in future animal studies.

Incidence of gallstone-induced acute cholecystitis is expected to increase with age 7 . However, elderly patients with fragility are unsuitable candidates for surgery. Thus, this single-step PS system may be suitable for long-term PS placement in the elderly, because EC-LAMS may be costly and is associated with bleeding and buried stent syndrome 8 . Furthermore, if a patient's performance status improves, elective laparoscopic cholecystectomy may become feasible. Previous reports indicate that EUS-GBD with LAMS does not prevent patients from eventually undergoing laparoscopic surgery 9 . However, pericholecystic adhesions and/or fistulas associated with LAMS have reportedly hindered laparoscopic procedures and necessitated open surgery in some cases 10 . Consequently, feasibility of laparoscopic cholecystectomy following EUS-GBD with LAMS remains under discussion. It is anticipated that affordable PSs may offer improved biocompatibility compared with LAMS.

This experimental and preliminary study of a single-step PS system during simulation of EUS-GBD used three artificial gallbladder models. Therefore, there is a need to proceed to the next stage of experimental animal studies. The appropriate degree of stent curling, adequate tip stiffness, and diameter of the electrocautery inner sheath, sufficient pushability against the walls, and optimal settings of the electrosurgical generator should be determined in the EUS-GBD animal model. Subsequently, Phase 1 clinical trials and additional studies should be conducted to obtain approval for commercialization. In the future, we anticipate well-designed comparative studies that will include EC-LAMS, current PS, and single-step PS systems.

## Conclusions

The newly developed PS system saved significant time, maintained intra-gallbladder pressure, and prevented bile leakage during the procedure when compared with the current PS system.
